# Metabolomics Analysis of the Spoilage Characteristics of *Pseudomonas fragi* and *Aeromonas salmonicida* Co-Culture in Refrigerated Grass Carp

**DOI:** 10.3390/foods14183228

**Published:** 2025-09-17

**Authors:** Yanlong Liu, Shuya Guo, Ruyan Xue, Li Liu, Abdul-Nabi Jatt, Caili Zhang

**Affiliations:** 1 School of Food Engineering, Ludong University, Yantai 264025, China17615357638@163.com (S.G.);; 2 Yantai Key Laboratory of Nanoscience and Technology for Prepared Food, Yantai Engineering Research Center of Green Food Processing and Quality Control, Yantai 264025, China; 3 Department of Food Science and Technology, National University of Singapore, Singapore 117542, Singapore; 4 Institute of Microbiology, University of Sindh, Jamshoro 76080, Pakistan

**Keywords:** spoilage, *Pseudomonas fragi*, *Aeromonas salmonicida*, metabolomic

## Abstract

*Pseudomonas fragi* and *Aeromonas salmonicida* are major spoilage microorganisms in refrigerated grass carp. This study systematically investigated the physicochemical and metabolomic characteristics of chilled grass carp that were artificially inoculated with *P. fragi* and *A. salmonicida* in mono- and co-culture. The results indicated that *P. fragi* was the dominant bacterium in the co-culture of grass carp. The *P. fragi*-inoculated group exhibited significantly higher levels of total volatile basic nitrogen and thiobarbituric acid reactive substances (TBARs, byproducts of lipid peroxidation) compared with the *A. salmonicida* group. Moreover, the TBAR levels were greater in the co-culture than in the *A. salmonicida* group at the end of storage. A metabolomic analysis revealed that 712, 424, and 465 differential metabolites were identified in grass carp inoculated with *A. salmonicida*, *P. fragi*, and their co-culture, respectively. The metabolic pathway enrichment showed that purine metabolism, aminoacyl-tRNA biosynthesis, glycerophospholipid metabolism, and amino acid metabolism were prevalent across all three inoculated groups. A total of 175 amino acids, peptides, and analogues were identified in the *A. salmonicida* group, indicating that *A. salmonicida* played a vital role in protein degradation. *P. fragi* was primarily enriched in linoleic acid metabolism and the biosynthesis of unsaturated fatty acids and fatty acids, demonstrating its advantages in lipid metabolism. Additionally, six potential spoilage biomarkers were identified, including inosine, cytidine, L-aspartic acid, L-tyrosine, Pro-Ile and PS(17:1(9Z)22:0). These results elucidated the complex and competitive interactions between *A. salmonicida* and *P. fragi* in the spoilage of grass carp, providing a scientific basis for the quality monitoring of grass carp and targeted preservation strategies.

## 1. Introduction

Grass carp (*Ctenopharyngodon idella*) is the most extensively cultivated freshwater fish species across China. In 2023, their total production reached 5,941,315 tons, accounting for nearly 20% of the country’s total volume of freshwater farmed fish [1]. Grass carp serves as a vital protein source and contributes significantly to China’s agricultural economy. The muscle tissue of grass carp, characterized by tenderness, a high moisture content, and abundant protein and unsaturated fatty acids, is particularly prone to quality deterioration due to microbial spoilage, endogenous enzyme activity, and lipid oxidation during storage. These factors can lead to texture softening, the accumulation of volatile odor compounds, and nutrient loss, ultimately resulting in spoilage [2]. Therefore, elucidating the contributions of microorganisms to fish spoilage is essential for driving innovations in preservation technology.

The major microorganisms responsible for fish spoilage are recognized as specific spoilage organisms, predominantly comprising *Pseudomonas* spp., *Aeromonas* spp., *Shewanella* spp., and *Acinetobacter* spp. [3,4]. Our previous study identified *Aeromonas salmonicida* and *Pseudomonas fragi* as the major spoilage microorganisms in refrigerated grass carp [5]. *A. salmonicida* is naturally present in the aquatic environment and is a pathogen known to cause furunculosis in fish, indicating its ubiquity in water sources [6]. *A. salmonicida* exhibits strong motility, which enables it to adhere to the fish’s surface. Additionally, it can secrete extracellular enzymes that decompose proteins and amino acids, resulting in the production of various spoilage metabolites, such as total volatile base nitrogen (TVB-N) and biogenic amines, which contribute to tissue degradation in fish and a decline in sensory quality [7,8]. *P. fragi*, as a typical psychrotrophic bacterium, is widely distributed in chilled fish and meat [9]. It can form biofilms at low temperatures, enhancing its ability to survive [10]. Furthermore, the production of extracellular enzymes and siderophores by *P. fragi* promotes its spoilage characteristics [11]. For grass carp, contamination by both bacteria mainly originates from the farmed water environment, cross-contamination during post-harvest handling, processing, and transportation, as well as from the fish’s surface, gills, and intestines at the time of slaughter [12]. Therefore, clarifying their spoilage mechanism is important for extending the freshness period of grass carp.

In actual food environments, multiple species of microorganisms usually coexist and interact with one another through competition, antagonism, and mutualism. These interactions can significantly alter spoilage characteristics compared with those observed with a single bacterial culture [13,14]. Prior research has indicated that multispecies interactions can influence food spoilage dynamics. For instance, the co-culture of *Brochothrix thermosphacta* and *P. fragi* significantly accelerated the spoilage rate of chilled pork [15]. Similarly, the co-culture of *P. fragi* and *Pseudomonas. lundensis* enhanced the production of histamine and putrescine [16]. These studies illustrate that when spoilage bacteria coexist in food, their growth and metabolism influence each other. The initial microflora of freshly caught grass carp is predominantly composed of *Pseudomonas spp*, *Aeromonas* spp, *Acinetobacter* spp, *Shewanella* spp, and *Bacillus* spp. [17]. Although the spoilage potential of single species such as *A. salmonicida* and *P. fragi* has been studied extensively, research on their interactions is still relatively limited.

Metabolomics is an emerging biological technique that characterizes changes in the metabolic products of cells. Due to its high sensitivity and resolution, it has been widely used to investigate the spoilage mechanism and identify biomarkers during food storage [18]. For instance, Dou et al. [19] discovered that propionic acid and L-phenylalanine could serve as potential biomarkers for spoilage in oysters through untargeted metabolomics. Additionally, Lou et al. [20] reported the identification of 39 metabolites in golden pomfret inoculated with *S. baltica* using nuclear magnetic resonance-based metabolomics. Hence, metabolomic technology is an effective method for uncovering the spoilage mechanisms of microorganisms during the storage of refrigerated grass carp.

This study aimed to explore the interaction between *P. fragi* and *A. salmonicida* in the storage of grass carp. The microbial enumeration and spoilage characteristics of grass carp in terms of TVB-N and thiobarbituric acid reactive substances (TBARs) were evaluated. Moreover, metabolomic technology was employed to further investigate the differences in spoilage between co-culture and mono-culture bacteria. This study can provide important theoretical support for elucidating the mechanism of spoilage caused by spoilage bacteria in aquatic products.

## 2. Materials and Methods

### 2.1. Bacterial Strains

The *P. fragi* and *A. salmonicida* used in this study were previously isolated from spoiled refrigerated grass carp. They were kept in our laboratory and were activated in Luria–Bertani (LB) agar at 30 °C overnight.

### 2.2. Preparation of Grass Carp

The live grass carp were obtained from a aquatic market in Yantai, Shandong, China. The sterile grass carp were prepared according to the method of Wang et al. [21]. Grass carp were stunned, scaled, and gutted. After washing, they were cut into 2 × 2 × 3 cm sticks. Then, the sticks were immersed in 75% ethanol for 5 min, subsequently they were washed with sterilized distilled water three times and dried under the ultraviolet radiator in a clean bench for 30 min.

The strains of *A. salmonicida* and *P. fragi* were pre-cultured in LB broth. Then, they were diluted in sterile physiological saline to obtain approximately ~10^5^ CFU/mL suspension. The sterilized fish sticks were randomly divided into four groups and immersed with (Ⅰ) *P. fragi* (PF group); (Ⅱ) *A. salmonicida* (AS group); (Ⅲ) a co-culture of *P. fragi* and *A. salmonicida* at a 1:1 ratio (Co-culture group); and (Ⅳ) sterile physiological saline (CK group) for 15 min, respectively. After that, the fish sticks were dried and packaged in polyethylene bags and then refrigerated at 4 °C for 12 d under aerobic conditions. Microbial enumeration, TVB-N and TBARs were analyzed at days 0, 2, 4, 6, 9, and 12. The texture of the fish sticks was determined at days 0, 4, 9 and 12.

### 2.3. Bacterial Enumeration

A 25 g sample of fish sticks was homogenized with 225 mL of sterile physiological saline. Serial ten-fold dilutions were prepared and 100 μL samples of the suspensions were spread onto plate count agar, incubated at 30 °C for 48 h, to determine the total viable count (TVC) [22].

### 2.4. Microbial Composition Determination

The samples from the co-culture group were prepared; 10 g of fish flesh from three independent packing bags was mixed and homogenized with 40 mL of sterile physiological saline. After centrifugation at 8000 rpm for 10 min, the precipitates were collected. The DNA was extracted using a TIANamp bacterial DNA extraction kit (Tiangen, Beijing, China). PCR amplification was performed in a reaction system containing 15 µL of Phusion^®^ High-Fidelity PCR Master Mix, 0.2 µM each of the 515F and 806R primers, and 10 ng of template DNA. The PCR amplification and library construction followed the method described in our previous study [23]. High-throughput sequencing was conducted using the NovaSeq6000 platform at Novegene Technology Co., Ltd. (Beijing, China).

### 2.5. Determination of TVB-N

TVB-N was quantified following the method reported by Cai et al. [24] using a Kjeldahl apparatus (Kjeltec 8400, Hilleroed, Denmark). The results were expressed as milligrams of TVB-N/100 g fish.

### 2.6. Determination of TBARs

The TBARs of fish flesh were measured using the method described by Chen et al. [25]. Briefly, 5.0 g of fish flesh was homogenized with 45 mL of 7.5% trichloroacetic acid for 2 min. The resulting mixture was then filtered through filter paper. Next, 2 mL of the filtrate was combined with 2 mL of TBARs solution (0.02 mol/L) and reacted at 90 °C for 40 min. After cooling, the absorbance of the samples was measured at 532 nm.

### 2.7. Texture Determination

Texture profile analysis was performed to determine the hardness, springiness, chewiness, and gumminess of the fish flesh according to the method of Lou et al. [26]. Briefly, the fish flesh was cut into 1 cm^3^ cubes and analyzed using a CT3–10k texture analyzer (Brookfield, Middleboro, MA, USA) with a P35 probe. The samples were compressed to 50% of deformation using a trigger force of 5 g. The pre-test speed, test speed and post-test speed were 1.0 mm/s, 2.0 mm/s and 2.0 mm/s, respectively.

### 2.8. Metabolomics Analysis

A 30 mg fish flesh sample was thoroughly ground with 600 μL of 80% (*v*/*v*) methanol solution and placed on ice for 1 h. After centrifugation at 12,000 rpm for 10 min (4 °C), 300 μL of supernatant was collected for LC-MS analysis. Six biological replicates of each group were independently processed for metabolomics analysis. An untargeted metabolomic analysis of grass carp extracts was performed using a Waters ACQUITY UPLC I-Class plus/Thermo QE plus system (Waters, Milford, MA, USA) with an ACQUITY UPLC HSS T3 column (1.8 μm, 100 × 2.1 mm). The mobile phase consisted of ultrapure water containing 0.1% (*v*/*v*) formic acid (phase A) and acetonitrile (phase B). A gradient elution program was employed as follows: 5% B (0 min), 30% B (4 min), 50% B (8 min), 20% B (10 min) and 100% B (14 min), followed by re-equilibration at 5% B for 2 min. Chromatographic separation was performed with a 3 μL injection volume and a flow rate of 0.35 mL/min at 45 °C. Mass spectrometric analysis was conducted in both positive and negative ionization modes with the following parameters: spray voltage of 3.8 kV (positive) and −3.0 kV (negative); capillary temperature of 320 °C; mass scan range of 100–1200 *m*/*z*; full MS resolution of 70,000; and stepped collision energies of 10, 20, and 40 eV [27].

Raw MS data were processed using metabonomics software Progenesis QI v3.0 (Nonlinear Dynamics, Newcastle, UK). The Human Metabolome Database, Lipidmaps (v2.3), METLIN, and the local LuMet-Animal 3.0 database were employed for compound identification [28].

### 2.9. Statistical Analysis

Microbiological and physicochemical analyses were conducted in triplicate. Untargeted metabolomics analysis included six biological replicates. Principal component analysis (PCA) and orthogonal partial least squares discriminant analysis (OPLS-DA) were employed to analyze the metabolic differences between groups. Data were analyzed via one-way analysis of variance using SPSS software (version 19.0). Statistics with *p* < 0.05 were considered significant.

## 3. Results and Discussion

### 3.1. Microbial Analysis

**Figure 1:**
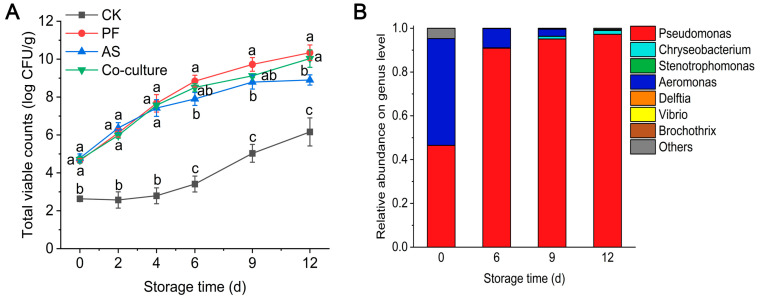
Microbial characteristics of refrigerated grass carp. (**A**) Changes in total viable count of refrigerated grass carp inoculated with *P. fragi*, *A. salmonicida*, and their co-culture during storage. (**B**) Microbial composition in refrigerated grass carp fillets in the co-culture group at genus level. Different letters represent significant differences (*p* < 0.05) between different groups at the same day. CK, PF, and AS represent control, *P. fragi*, and *A. salmonicida*, respectively.

The changes in the TVC of grass carp inoculated with *P. fragi*, *A. salmonicida* and their combination are shown in Figure 1A. The initial TVCs in the *P. fragi*, *A. salmonicida* and co-culture groups were 4.65, 4.78 and 4.70 log CFU/g, respectively, aligned with fresh grass carp fillets [29]. The CK group had a low initial TVC (2.63 log CFU/g), which was significantly lower than that of the inoculation groups (*p* < 0.05), meeting the requirement for sterile fish fillets. The TVC increased rapidly in all inoculated groups, and no significant differences were observed during early storage (0~4 d) (*p* > 0.05), suggesting that both bacteria adapted readily. However, the *P. fragi* group exhibited significantly faster growth than *A. salmonicida* from day 6 onwards (*p* < 0.05). On day 12, TVC reached 10.03 log CFU/g for *P. fragi*, significantly higher than *A. salmonicida* (8.90 log CFU/g) (*p* < 0.05), indicating *Pseudomonas* spp.’s superior growth and adaptability in refrigerated grass carp. This phenomenon may be attributed to its greater psychrotolerance and nutrient utilization efficiency compared with *Aeromonas* spp. [30,31]. The TVC in the co-culture group did not differ significantly from the single-species groups at day 6 and day 9 (*p* < 0.05), suggesting their coexistence without apparent interaction at this stage. After 12 days of storage, the TVC in the co-culture group matched the *P. fragi* group and significantly exceeded the *A. salmonicida* group, demonstrating *P. fragi*’s eventual dominance. These results suggest that *P. fragi* gradually became dominant in the co-culture system, which is attributed to the stronger cold adaptability and efficient proliferation of *Pseudomonas* spp. during prolonged refrigeration. These findings were consistent with reports that *Pseudomonas* spp. is the dominant spoilage bacteria in refrigerated grass carp [29,32].

The change in the microbial composition of the co-culture group is shown in Figure 1B. Initially, the microbial composition of grass carp was predominantly made up of *Pseudomonas* spp. and *Aeromonas* spp., which constituted 46.5% and 48.8% of the total, respectively. With an increase in storage time, *Pseudomonas* spp. became the dominant species, with its proportion increasing to 90.9% on day 6, while the proportion of *Aeromonas* spp. declined to 8.8%. This shift indicated that *Pseudomonas* spp. has a competitive growth advantage in the co-culture system. The decline of *Aeromonas* spp. is due to its competitive disadvantages in acquiring nutrients or the negative effects of metabolic products from *Pseudomonas*. Fang et al. [33] reported that the population of *P. lundensis* reached 91.6% at the end of beef storage when co-cultured with *B. thermosphacta*, in accordance with our observation. Similarly, *P. fluorescens* also exhibited an exceptional capacity for growth in the co-culture with *S. putrefaciens* [16]. These findings support the conclusion that psychrotrophic *Pseudomonas* species are critical microorganisms in aerobically stored chilled high-protein foods [34].

### 3.2. TVB-N Analysis

**Figure 2:**
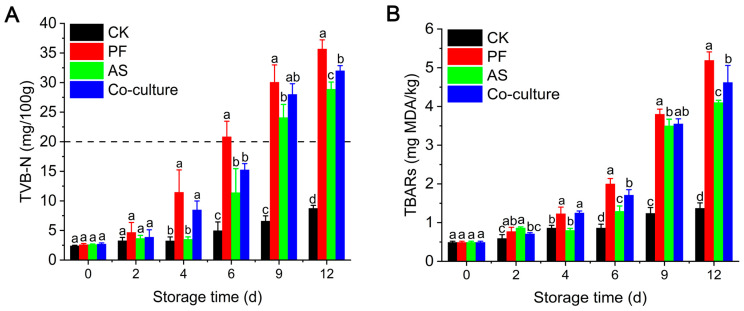
The change in TVB-N (**A**) and TBARs (**B**) of different groups during storage at 4 °C. Different letters represent significant differences (*p* < 0.05) between different groups at the same day. CK, PF, and AS represent control, *P. fragi*, and *A. salmonicida*, respectively.

TVB-N, which comprises alkaline nitrogenous compounds including ammonia and amines, is a critical freshness indicator for aquatic products [35]. As illustrated in Figure 2A, compared with the CK group, the TVB-N levels in the three bacterial inoculated groups increased rapidly as the storage time extended, indicating that microbial activity is the primary driver of increases in TVB-N. From day 4 onward, the *P. fragi* group showed significantly higher TVB-N levels compared with the *A. salmonicida* and co-culture groups (*p* < 0.05), highlighting the important role of *P. fragi* in TVB-N production. Similarly, Wang et al. [36] and Ge et al. [37] also demonstrated that *P. fragi* showed a significant TVB-N production capacity in yellow catfish and large yellow croaker. Specifically, the TVB-N level in the *P. fragi* group exceeded China’s regulatory limit of 20 mg/100 g for freshwater fish (GB 2733-2015 [38]), reaching 20.84 mg/100 g at day 6, which was 11.39 mg/100 g and 15.25 mg/100 g in the *A. salmonicida* and co-culture groups, respectively. These results suggested that the potential TVB-N production of *A. salmonicida* was weaker than that of *P. fragi*. TVB-N generally originates from the degradation of proteins and other nitrogen (N)-containing compounds due to the activity of spoilage bacteria [39]. The rapid accumulation of TVB-N in the *P. fragi* group was mainly attributed to its strong adaptation to low-temperature environments or its high proteolytic activity [40]. Liu et al. [41] discovered a protease named AprA from *P. fragi*, which degraded myofibrillar and sarcoplasmic proteins, leading to an increase in TVB-N levels. Notably, the accumulation of TVB-N in the co-culture group was higher than that of the *A. salmonicida* mono-culture at the end of refrigeration storage; this was attributed to the predominance of *Pseudomonas* spp., consistent with the results of microbial proliferation.

### 3.3. TBARs Analysis

Lipid oxidation serves as a vital factor that contributes to the deterioration of fish quality. Malondialdehyde (MDA), one of the products of lipid oxidation, can react with thiobarbituric acid to form a red complex [42]. In grass carp, the muscle lipid content is approximately 3.3% of the wet weight, making it highly susceptible to oxidation and hydrolysis due to bacterial action and exposure to air [43]. Figure 2B shows that the initial TBARs value in grass carp was 0.49 mg MDA/kg fish. The CK group showed a slight increase in TBARs, while those in the groups inoculated with bacteria increased rapidly as the storage time prolonged. The *P. fragi* group exhibited a significantly higher TBARs value than the *A. salmonicida* group on day 4 (*p* < 0.05), which might be due to the strong lipase activity in *P. fragi*. The dominant lipids in grass carp tissue include phosphatidylcholine (PC), triglyceride (TG) and phosphatidylethanolamine (PE) [44]. These lipids can be hydrolyzed by phospholipases and lipases. Ge et al. [37] reported that *P. fragi* strains isolated from large yellow croaker exhibited potent extracellular lipase activity, supporting its role in lipid oxidation and the deterioration of quality.

### 3.4. Changes in Texture of Fish

**Figure 3:**
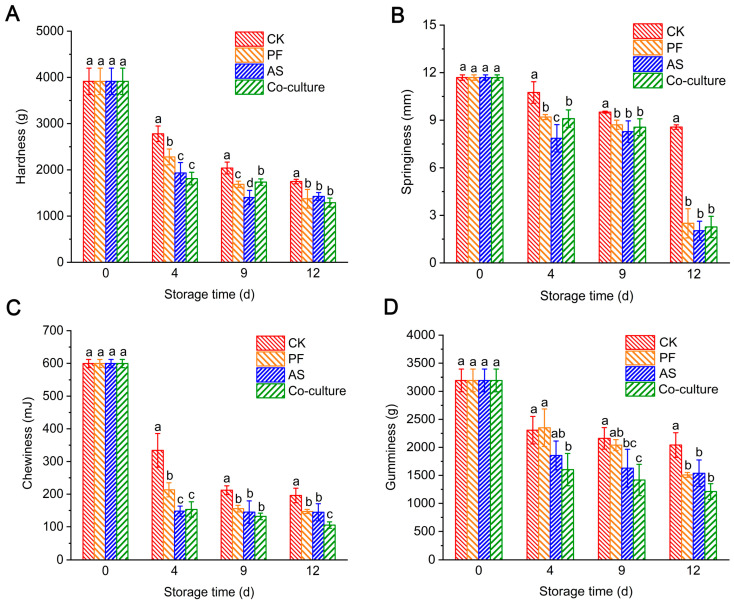
The change in hardness (**A**), springiness (**B**), chewiness (**C**), and gumminess (**D**) of different treatment groups during storage at 4 °C. Different letters represent significant differences (*p* < 0.05) between different groups at the same day. CK, PF, and AS represent control, *P. fragi*, and *A. salmonicida*, respectively.

The changes in the texture of fish flesh inoculated with different bacteria are shown in Figure 3. The hardness of the fish flesh in the CK group was 3914.17 g at day 0, and it decreased to 1747.33 g after 12 days of storage (Figure 3A). In the inoculated groups, the hardness decreased more rapidly, dropping to 1290.00–1374.67 g. The decrease in hardness was attributed to the action of endogenous protease and extracellular protease produced by microorganisms. These proteases hydrolyze fish protein, leading to the rupture of muscle segments and the loosening of the myofibrillary structure, which ultimately results in a softer muscle texture [26]. Notably, the *A. salmonicida* group exhibited significantly reduced hardness compared with the *P. fragi* group at days 4 and 9 (*p* < 0.05), suggesting more pronounced protein degradation by *A. salmonicida*. This enhanced degradation may be due to the strong protease activities exhibited by *A. salmonicida*. Our previous study showed that *Aeromonas* spp. has stronger protease activity than *Pseudomonas* spp. [5]. Similarly, Shao et al. [45] found that the extracellular protease Hap from *A. salmonicida* can hydrolyze collagen, resulting in a decline in the quality of chicken. Myofibrillar protein determines the contractility of muscles and affects the texture characteristics of fish products, such as their hardness, springiness, chewiness and gumminess. Similarly, the springiness, chewiness and gumminess in the three inoculated groups declined dramatically. Texture profiling revealed significantly lower springiness and chewiness in *A. salmonicida*-inoculated samples compared with the *P. fragi* group at day 4 (*p* < 0.05) (Figure 3B), indicating that *A. salmonicida* proliferates rapidly and secretes substantial extracellular proteases during initial storage. At the end of storage, the springiness values converged across all bacterial inoculation groups, indicating that microbial activity was the primary reason for the degradation of the texture of grass carp. During late-stage storage, bacterial proliferation accelerates spoilage and the breakdown of fish tissue. This proteolytic activity disrupts the myofibrillar structure, leading to tissue softening and significant reductions in textural parameters such as chewiness and gumminess [46].

### 3.5. Metabolomic Analysis

#### 3.5.1. Multivariate Analysis

**Figure 4:**
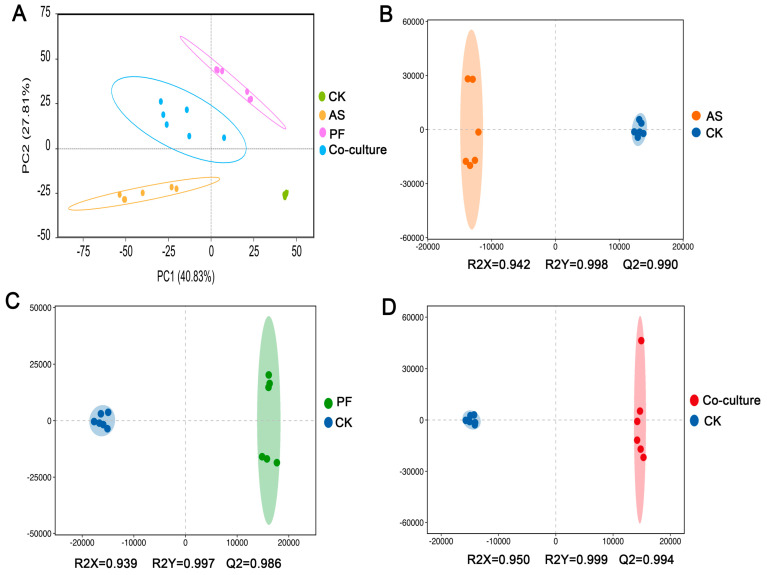
PCA analysis of grass carp in different treatment groups after 9 days of storage time (**A**). OPLS-DA analysis between *A. salmonicida* and CK groups (**B**), *P. fragi* and CK groups (**C**), and co-culture and CK groups (**D**). CK, AS, and PF represent control, *A. salmonicida*, and *P. fragi*, respectively.

Untargeted metabolomics was employed to characterize variations in the metabolite profiles of fish flesh among various groups during refrigerated storage. High-resolution mass spectrometry detected 6009 metabolites, including 2791 in positive ionization mode and 3218 in negative ionization mode (Table S1). A plot of the PCA scores showed the global metabolic differences among different treatment groups [47]. Figure 4A indicates that PC1 explained 40.83% of the total variance and that PC2 contributed an additional 27.81%. The four groups were separated clearly and the six biological replicates within each group clustered closely, demonstrating high repeatability and data reliability.

OPLS-DA, a supervised multivariate technique, is usually employed to remove orthogonal variation unrelated to class discrimination [48]. Model robustness is evaluated using R^2^Y and Q^2^, which denote the goodness of fit and predictive capacities of the model, respectively. OPLS-DA was employed to evaluate the relationships between *A. salmonicida*, *P. fragi*, and their co-culture relative to the CK group. Figure 4B revealed that the AS and CK groups were clearly separated, with R^2^Y = 0.998 and Q^2^ = 0.990, indicating significant metabolic differences and a reliable predictive capability. Similar distinctions were also observed between the PF and CK groups (Figure 4C), as well as between the co-culture and CK groups (Figure 4D), demonstrating the effectiveness of the OPLS-DA model.

#### 3.5.2. Differential Metabolites Analysis

**Figure 5:**
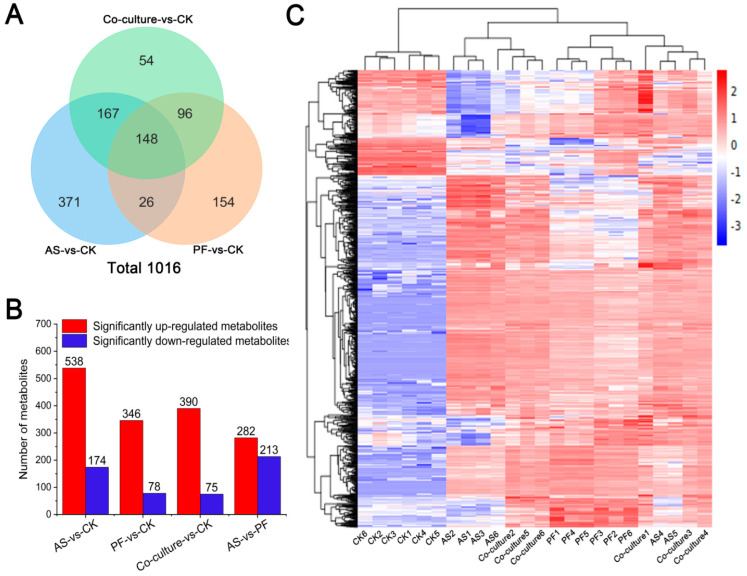
Venn diagram for the four groups of comparison (**A**). The number of differential metabolites among different groups (**B**). Heatmap of the 1016 different metabolites in different treatment groups of refrigerated grass carp (**C**). CK, AS, and PF represent control, *A. salmonicida*, and *P. fragi*, respectively.

The variable importance of the projection (VIP) value for each metabolite was derived from the OPLS-DA model. Differential metabolites among the four groups were identified through rigorous screening criteria including VIP > 1, *p* < 0.05, and fold change (FC) ≥ 1.2 or ≤ 0.833. Comparative analysis revealed 1016 differential metabolites across all groups (Figure 5A, Table S2), which comprised 365 lipids and lipid-like molecules, 226 organic acids and derivatives, 96 organoheterocyclic compounds, 55 organic oxygen compounds, 49 benzenoids, and 225 metabolites distributed among other biochemical classes. To more intuitively illustrate the differences in the expression of metabolites across the various groups, hierarchical clustering was conducted with the 1016 differential metabolites; the results are shown in Figure 5C. This analysis revealed distinct metabolic profiles between the bacterial inoculation groups and the CK group, indicating that microbial activity significantly contributes to the production of spoilage metabolites in grass carp.

**Figure 6:**
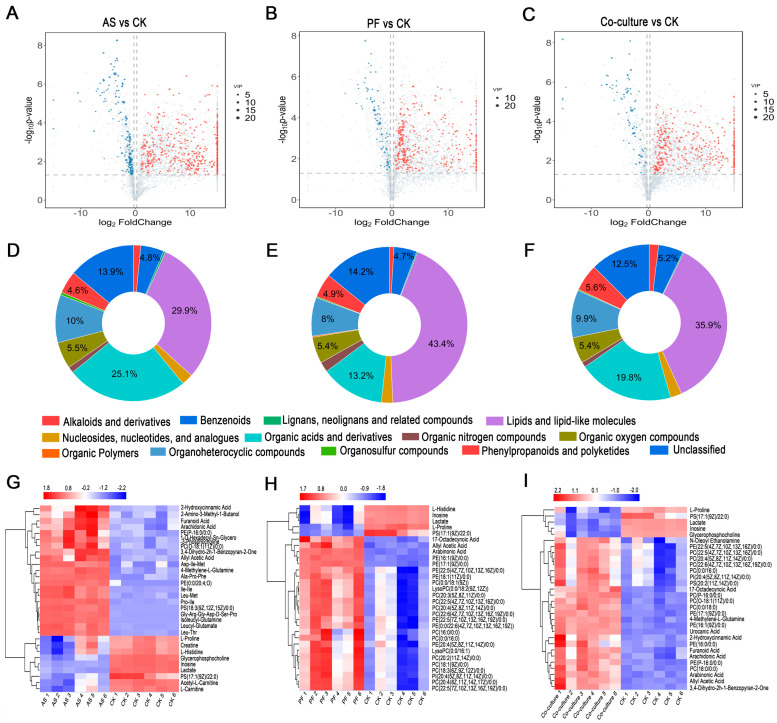
Volcano plots of differential metabolites between *A. salmonicida* and CK groups (**A**), *P. fragi* and CK groups (**B**), and co-culture and CK groups (**C**). Compositions of metabolic types between *A. salmonicida* and CK groups (**D**), *P. fragi* and CK groups (**E**), and co-culture and CK groups (**F**). Heatmap of clustering hierarchical analysis of the top 30 differential metabolites between *A. salmonicida* and CK groups (**G**), *P. fragi* and CK groups (**H**), and co-culture and CK groups (**I**). CK, AS, and PF represent control, *A. salmonicida*, and *P. fragi*, respectively.

The differential metabolites were analyzed in the paired comparison groups of AS vs. CK, PF vs. CK, and co-culture vs. CK. As shown in Figure 5B and Figure 6A, 712 differential metabolites were identified in the AS vs. CK comparison, including 538 upregulated and 174 downregulated metabolites, suggesting the strong metabolic activity of *A. salmonicida*. Among the top 30 differential metabolites presented in Figure 6G, 10 metabolites belonging to small peptides were significantly upregulated. Notably, Ala-Pro-Phe, Asp-Ile-Met, and Ile-Ile exhibited substantial increases (12.99, 12.12, and 11.21 log_2_FC, respectively), strongly indicating protein degradation in fish inoculated with *A. salmonicida*. This finding aligned with previous reports that *Aeromonas* spp. can secrete various extracellular proteases, which degrade food proteins and release small peptides [49]. Supporting this mechanism, Shao et al. [50] identified 16 proteases from *A. salmonicida* that are associated with spoilage using proteomics technology. Liu et al. [51] also found six metalloproteases and seven serine proteases genes in the *A. salmonicida* GMT3 genome. These results provide a material basis for its protein degradation. Proline showed −1.12 log_2_FC downregulation, inferring that proline may be one of the crucial substrates for maintaining the growth of *A. salmonicida* [52].

The PF vs. CK comparison revealed 424 differential metabolites, including 346 upregulated and 78 downregulated metabolites (Figure 5B and Figure 6B). Among the top 30 differential metabolites (Figure 6H), 21 metabolites belonged to glycerophospholipids; among these, PE(16:1(9Z)/0:0) and PE(17:1(9Z)/0:0) were significantly upregulated, with 6.54 and 9.38 log_2_FC, respectively, suggesting that cell membrane phospholipids were among the main targets for *P. fragi*. Xin et al. [53] and Ge et al. [37] have reported that *P. fragi* exhibits strong lipase activity. Therefore, *P. fragi* may contribute to fish spoilage by secreting lipase, which leads to lipid oxidation, consistent with the above TBARs results. Previous studies have indicated that when protease activity in *Pseudomonas* decreases, lipase activity tends to increase [54]. A similar pattern was observed in this study. Chang et al. [55] investigated the enzyme production capacity of 106 strains of *Pseudomonas* spp., and the results indicated that 50% of these strains can secret protease, with 83.02% having the ability to produce lipase. Additionally, L-Histidine showed −3.15 log_2_FC downregulation, which may be related to its utilization by *P. fragi* for histamine formation.

A total of 390 differential metabolites were upregulated, while 75 metabolites were downregulated between the co-culture and CK groups (Figure 5B and Figure 6C). Among the top 30 differential metabolites, seventeen metabolites belonged to glycerophospholipids (Figure 6I); these primarily originated from *P. fragi*, which dominated at the end of the storage. Urocanic acid, an important intermediate in histidine metabolism [56], exhibited 6.58 log_2_FC upregulation in the co-culture group. This increase can be attributed to the metabolic activity of *P. fragi*, as this organism showed a significant decrease in histidine levels.

#### 3.5.3. Metabolic Pathway Analysis

**Figure 7:**
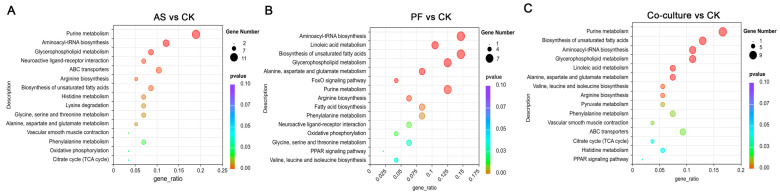
KEGG enrichment pathway analysis between *A. salmonicida* and CK groups (**A**), *P. fragi* and CK groups (**B**), and co-culture and CK groups (**C**). CK, AS, and PF represent control, *A. salmonicida*, and *P. fragi*, respectively.

Pathway enrichment analysis via the KEGG database revealed significant differences in metabolic pathways, with the top 15 enriched pathways displayed in Figure 7. Notably, purine metabolism emerged as the most significant enriched pathway across all bacterial inoculation groups, highlighting the crucial role of microbial activities in purine metabolism. Purine metabolites serve as essential flavor determinants in fish, with distinct nucleotides contributing specific taste profiles. Inosine monophosphate (IMP), the predominant umami-enhancing nucleotide in fresh fish, acts as the primary flavor modulator, while adenosine monophosphate (AMP) imparts a sweet taste. In contrast, hypoxanthine and xanthine are known for their bitter taste and off-flavor, which are typically linked to fish spoilage [57]. In this study, AMP was significantly downregulated in all three bacterial inoculation groups, suggesting that spoilage bacteria accelerated AMP degradation. Frank et al. [58] have reported that AMP is an intermediate in the metabolism of adenosine triphosphate (ATP) and can serve as an indicator of ongoing bacterial metabolism in meat products. Additionally, the abundance of inosine was reduced, while there was an increase in the metabolites of xanthine and guanine, indicating that microbial activities enhanced purine metabolism. Previous studies reported that spoilage bacteria influenced the conversion of hypoxanthine to xanthine [59,60]. Notably, the role of *A. salmonicida* in the purine metabolism of refrigerated grass carp was more pronounced than that of *P. fragi*. Specifically, the abundance of inosine was downregulated by −3.73 log_2_FC in the *A. salmonicida* group and −2.13 log_2_FC in the *P. fragi* group, while the abundance of xanthine was upregulated by 2.29 log_2_FC in the *A. salmonicida* group and 1.15 log_2_FC in the *P. fragi* group. Chen et al. [25] also reported that *A. salmonicida* had a strong ability to degrade hypoxanthine into xanthine. Furthermore, no significant differences in the levels of inosine and xanthine were observed between the co-culture and *A. salmonicida* groups. Adenosine, the ATP degradation product, was significantly upregulated only in the *A. salmonicida* and co-culture inoculation groups, indicating that purine metabolism in the co-culture group was primarily driven by *A. salmonicida*.

Aminoacyl-tRNA biosynthesis was significantly enriched in all three inoculated groups, indicating a high dependence on protein synthesis and the amino acid metabolism of spoilage bacteria. ATP-binding cassette (ABC) transporters, which are crucial for nutrient uptake and drug resistance [61], were significantly enriched in the *A. salmonicida* group (Figure 7A). In combination with the results from differential metabolites analysis, 175, 47, and 83 kinds of differently expressed amino acids, peptides, and analogues (belonging to organic acids and derivatives) were identified in the *A. salmonicida*, *P. fragi*, and co-culture groups, respectively. The organic acids and derivatives compounds in these groups accounted for 25.1%, 13.2%, and 19.8% of the total compounds, respectively (Figure 6D–F). It was inferred that the spoilage mode for *A. salmonicida* is primarily driven by protein degradation, which can be attributed to its strong capacity for protein lysis. Our previous study demonstrated that *Aeromonas* exhibits stronger proteolytic activity, as evidenced by the clearer zones of transparency observed on skim milk agar plates [5]. Similarly, Zhuang et al. demonstrated that *Aeromonas* was more effective in hydrolyzing grass carp fish proteins through LC-MS/MS-based peptidomics [62].

In the PF vs. CK comparison, significant enrichment was observed in linoleic acid metabolism, the biosynthesis of unsaturated fatty acids, fatty acid biosynthesis, and glycerophospholipid metabolism, all of which were classified as lipid metabolism (Figure 7B). These findings suggested that the spoilage mechanism of *P. fragi* in grass carp primarily involved lipid oxidation and phospholipid degradation. Consistently, lipids and lipid-like molecules accounted for 29.9%, 43.4%, and 35.9% in the *A. salmonicida*, *P. fragi*, and co-culture groups, respectively (Figure 6D–F). These results indicated that *P. fragi* preferentially degraded fish lipids through the oxidation of unsaturated fatty acids, such as linoleic acid. The substantial production of glycerophospholipids reflected the high activity of bacterial lipase, which decomposed phospholipids in fish muscle cell membranes and released free fatty acids. Ge et al. [37] also reported that the *P. fragi* isolated from large yellow croaker exhibited strong lipase activity. The robust lipid metabolism activity of *P. fragi* is of great significance for its adaptation to low temperatures, which may be one of the primary reasons it became the dominant spoilage bacterium in refrigerated grass carp.

Amino acid metabolism is a core pathway for microbial growth and the production of spoilage metabolites. As illustrated in Figure 7, alanine, aspartate and glutamate metabolism, phenylalanine metabolism, and arginine biosynthesis were enriched in all three inoculated groups. Succinic acid, an intermediate product of the tricarboxylic acid (TCA) cycle [63], demonstrated increased abundance in *A. salmonicida*, *P. fragi*, and the co-culture groups, showing 4.53, 4.88, and 6.99 log_2_FC upregulation, respectively. These findings indicated that both bacteria derived energy for their growth through the TCA cycle, with a synergistic effect observed in the co-culture group. Glutamine plays a crucial role in nitrogen metabolism [64]. The glutamine levels in the *A. salmonicida* and *P. fragi* groups showed −1.74 and −5.94 log_2_FC downregulation, respectively, suggesting a higher nitrogen demand in *P. fragi*, which probably supported its rapid growth. In terms of histidine metabolism, L-histidine was downregulated by −1.05 log_2_FC in the *A. salmonicida* group and −3.15 log_2_FC in the *P. fragi* group, and its downstream products, formiminoglutamic acid and urocanic acid, showed 8.33 and 6.35 log_2_FC upregulation in the *P. fragi* group and 7.34 and 4.44 log_2_FC upregulation in the *A. salmonicida* group. These results indicated that *P. fragi* possessed greater histidine catabolic activity, aligned with the results of a previous study by Zhuang et al. [62], who demonstrated that *Pseudomonas* possessed greater amino acid metabolic activity than *Aeromonas* in refrigerated grass carp. Regarding L-threonine metabolism, *A. salmonicida* and *P. fragi* displayed opposing metabolic trends. Threonine and its derivatives, L-cystathionine and creatine, decreased in the *A. salmonicida* group but increased in the *P. fragi* group. This discrepancy may be attributed to the metabolic differences between the bacterial species. The co-culture group exhibited no significant enrichment of differential metabolites in threonine metabolism, indicating that the opposing metabolic differences between the two bacteria were balanced in the co-cultured system. Additionally, a decrease in creatine was observed in the *A. salmonicida* group, which may impact muscle energy metabolism and is associated with texture softening. These findings aligned with the observed decrease in texture in the *A. salmonicida* group. The results regarding amino acid metabolism were supported by Zhuang et al. [62], who indicated that spoilage in grass carp caused by spoilage bacteria was primarily related to amino acid metabolism.

#### 3.5.4. Potential Spoilage Biomarkers

**Figure 8:**
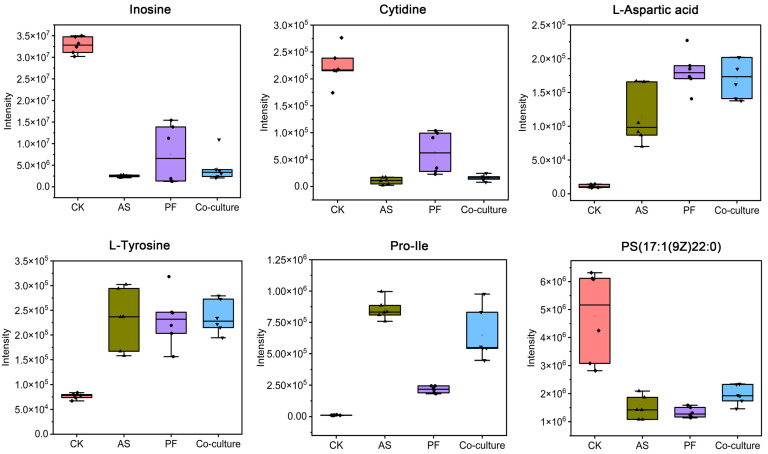
Relative changes of six potential spoilage biomarkers in refrigerated grass carp among *A. salmonicida*, *P. fragi*, co-culture and CK groups. CK, AS, and PF represent control, *A. salmonicida*, and *P. fragi*, respectively.

In this study, six key metabolites were identified as potential spoilage biomarkers in grass carp: inosine, cytidine, L-aspartic acid, L-tyrosine, Pro-Ile and PS(17:1(9Z)22:0) (Figure 8). Inosine and cytidine play a vital role in nucleic acid metabolism [65]. The abundance of both metabolites was consistently downregulated in the bacterial inoculation groups, indicating a decline in fish quality. Similarly, Fang et al. [33] also observed a decrease in inosine levels in the bacterial inoculated groups of refrigerated beef. The increase in L-aspartic acid, L-tyrosine and Pro-Ile suggests the degradation of fish protein [50]. The phospholipid PS(17:1(9Z)/22:0) is a key structural component of cellular membranes. It undergoes microbial degradation during spoilage, liberating free fatty acids that serve as sensitive indicators of quality deterioration in aquatic products. These six identified metabolites exhibited significant correlations with spoilage, suggesting their potential use as candidate biomarkers for monitoring the freshness of grass carp during refrigerated storage. This panel of metabolites provides specific molecular targets for developing novel preservation methods tailored to grass carp. Additionally, these markers could aid in the creation of rapid analytical methods to dynamically assess the deterioration of fish quality.

## 4. Conclusions

This study revealed the distinct metabolic profiles of *A. salmonicida* and *P. fragi* in both mono-culture and co-culture systems through metabolomics analysis. In co-culture, *P. fragi* demonstrated rapid growth through the enhanced biosynthesis of unsaturated fatty acids and fatty acids, ultimately dominating the microbial community (exceeding 90% abundance after 6 days of storage). Conversely, *A. salmonicida* exhibited superior proteolytic activity, evidenced by the significant accumulation of small peptides. Furthermore, six potential spoilage biomarkers, including inosine, cytidine, L-aspartic acid, L-tyrosine, Pro-Ile and PS(17:1(9Z)22:0), were identified. In the co-culture mode, the synergistic spoilage mechanism of grass carp involved coordinated action through purine metabolism, lipid metabolism, protein degradation, and amino acid metabolism. These findings will provide essential insights into bacterial interactions during the refrigerated storage of aquatic products. They will also establish a theoretical basis for monitoring the quality of aquatic products and for developing new targeted preservation strategies.

## Data Availability

The original contributions presented in the study are included in the article and Supplementary Materials, further inquiries can be directed to the corresponding author.

## Supplementary Materials

The following supporting information can be downloaded at: https://www.mdpi.com/article/10.3390/foods14183228/s1, Table S1: The detected 6009 metabolites of grass carp in different treatment groups; Table S2: The 1016 differential metabolites from four groups.

## Abbreviations

TVB-N	Total Volatile Basic Nitrogen
TBARs	Thiobarbituric Acid Reactive Substances
